# Quality of Erector Spinae Plane Block Educational Videos on a Popular Video-sharing Platform

**DOI:** 10.7759/cureus.4204

**Published:** 2019-03-07

**Authors:** Alessandro De Cassai, Christelle Correale, Ludovica Sandei, Irina Ban, Onur Selvi, Serkan Tulgar

**Affiliations:** 1 Anesthesiology, University of Padova, Padova, ITA; 2 Anesthesiology, University of Padova, Padova , ITA; 3 Anesthesiology, Maltepe University Faculty of Medicine, Istanbul, TUR

**Keywords:** education, anesthesia, internet, erector spinae plane block

## Abstract

Introduction

The Erector Spinae Plane (ESP) block is a novel inter-fascial block developed in 2016, which has several clinical indications. YouTube (www.youtube.com; YT) is a popular American video-sharing platform. YT permits every user to view, share, and comment the videos uploaded by other users. The aim of the study was to evaluate the educational value and the technical video quality of didactic videos for the ESP block on a popular video-sharing platform, to evaluate the difference in quality between academic and nonacademic videos, and to evaluate the correlation between the views and quality of the videos, the length, and the time since upload.

Methods

We performed a search on YT trying to detect all relevant educational videos for the ESP block. Both the educational value and the technical video quality were assessed independently by three assessors. Assessors were asked to watch the videos and to fill in two questionnaires, the first one regarding the technical and educational aspects of ESP block, the second one regarding the video-editing quality. The video length, academic origin, views, and time from upload were also registered.

Results

We identified 62 videos and 21 videos entered the final analysis. The educational material for the ESP block had an overall low quality. Academic videos have a higher quality than non-academic ones. The video views are correlated with time since upload but not with the video quality.

Conclusions

The educational material for the ESP block on YT has a limited technical and video quality. For this reason, we recommend physicians to be very cautious in using video-sharing platforms as a unique source of medical knowledge.

## Introduction

The Erector Spinae Plane (ESP) block is a novel inter-fascial block developed in 2016 which has several clinical indications varying from hip surgery to carotid endarterectomy [1-2]. It consists of an injection of a local anesthetic between erector spinae muscle group and transverse process of the underlying vertebra. It is an easy and safe block to perform for which sporadic complications were described [3-4]. However, because of its novelty, many physicians are not aware of it or are unable to perform it.

YouTube (www.youtube.com; YT) is a popular American video-sharing platform. YT permits every user to view, share, and comment the videos uploaded by other users. It has been shown in many studies that video-assisted learning of a procedure in a step-by-step manner can be more effective than the conventional didactic training methods [5]. This is one of the reasons that explains why video-sharing platforms have become very popular among patients and health professionals as a source of medical knowledge and as an educational tool. However, since no quality standards are imposed by the platform administrators to uploading users and videos undergo no peer review process before publication, this leads to mixed results of the reliability of the information and quality of the technical and educational aspects of the video offered to users.

The aims of this study were to a) evaluate the technical and video quality of the educational videos for the ESP block on a popular video-sharing platform, b) evaluate if there is a quality difference between academic and nonacademic videos, and c) evaluate if the video views increase according to quality, length, or time since upload.

## Materials and methods

The search was performed in a single session on December 26th, 2018 by one of the authors (ADC) on the video sharing platform YT. Search terms included the following terms: “Erector Spinae Plane Block” OR “ESP block” OR “Erector Spinae block”. We did not apply any restrictions on publication period in our search. A preselection was performed during the search excluding videos with a non-related title and videos lasting more than twenty minutes. The excluded videos lasting more than twent minutes to avoid lowering the accuracy of the assessment due to the excessive length.

Lenght(s), academic origin (yes/no), views (n), time from upload (months) and Uniform Resource Locators (URL) of remaining eligible videos were inserted in a database and shared with other investigators. “Academic videos” were defined as videos supported by an anesthesia society or videos produced by researchers with published papers on ESP block [6].

All videos were then examined by all investigators and excluded if a) content was not pertinent or if b) video language was other than English or if c) the ESP block was performed without ultrasound. The remaining videos were considered eligible for evaluation by three assessors (ADC, CC, LS).

Three investigators (ADC, CC, LS), experienced in interfascial blocks, were asked to watch the videos and to fill in two questionnaires. The first questionnaire (Table 1) included 14 questions aiming to evaluate the technical aspects of the ESP block, particularly the sterility, ultrasound technique, anatomy knowledge, indications and local anesthetic usage (from now on “technical quality”). First questionnaire originates from previous similar studies [7-8].

**Table 1:** First questionnaire

#	Description
1	Were the clinical indications for ESP block clearly explained?
2	Were anatomical landmarks clearly explained or marked?
3	Was the erector spinae muscle anatomy clearly explained?
4	Were the suspected mechanism of actions clearly explained?
5	Was the technical information for probe selection and frequency regarding the ultrasound device explained?
6	Was the ultrasound anatomy showed and explained clearly?
7	Were the sono-anatomic images recording and anatomical structures in the recording clear and easy to perceive?
8	Was the ultrasound image of the needle visible and easy to follow?
9	Were the instructions for depth, alignment and directional movements of the needle clearly explained?
10	Was information about the local anesthetic spread explained?
11	Was information about in-plane or out-plane technique presented in the video?
12	Was sterile technique clearly explained or emphasized?
13	Was information about local anesthetic substance clearly explained?
14	Were possible complications related to this block technique explained?

The second questionnaire (14 elements; Table 2) was about the technical aspects of an educational video making such as sound, images, step by step procedure, clearly showed key-points. The assessors evaluated the videos according to the Guidelines for the Preparation and Evaluation of Video Career Media by the American National Career Development Association (NCDA; from now on “video quality”) [9].

**Table 2:** Second questionnaire

#	Description
1	Was the aim of video clearly stated and was it explained in the first quarter of the video?
2	Did the title or name of the video match the aim of the video?
3	Were the design and the content of the video suitable for a targeted educational aim?
4	Were the skills and the technique of the procedure explained using a standard, comparable and “step by step” method?
5	Was the information given in the video useful for viewers to develop/enhance their skill base?
6	Was the content of the video appropriate for the health and safety of both the patient and the practitioner?
7	Was the quality of picture regarding colors and clarity acceptable?
8	Was the quality of video sound acceptable? (No sound should be scored as zero)
9	Was the length of the video in balance with the content of the video?
10	Was the information on the date of production or release, producers and the references clearly explained?
11	Were objectives, learning tasks and terminology clearly stated in the video enabling viewers to address those tasks?
12	Did the video have stop-and-discuss points, additional aids such as scripts and/or summarized information on procedure?
13	Was any information given on a way to evaluate the effectiveness and reproducibility of the video?
14	Did the content of the video stimulate viewers to make the transition from passive viewer to active practitioner in the application of the technique?

The assessors were asked to evaluate each question with a scale ranging from zero to five (zero: totally unsatisfying one: unsatisfactory, two: poor, three: satisfactory, four: good, five: outstanding). The sum of the median values for each question formed the final scores ranging between zero and 70. The final scores were grouped as follows: from zero to 13: unsatisfactory; from 14 to 27: poor; from 28 to 41: satisfactory; from 42 to 54: good; from 56 to 70: outstanding.

Statistical analysis

The normality of distribution of the quantitative variables was analyzed using the Shapiro-Wilk test. The normally distributed variables were compared using the two-tail Student’ t-test or the Mann-Whitney U test for non-normally distributed. Continuous variables are presented as mean ± standard deviation (SD) and 95% confidence interval (CI). The median and interquartiles range values are reported for non-normally distributed variables. To determine the strength and the direction of the association between two variables, we used the Bravais-Pearson’s correlation test for variables with a normal distribution and Spearman's rank correlation test for variables that did not meet the assumptions of a normal distribution. All statistical analyses were conducted using R version 3.4.0 (2017-04-21). *P*-values <0.05 were considered statistically significant.

## Results

Sixty-two videos have been identified; 22 videos which did not include the ESP block and four videos longer than 20 minutes were excluded at the pre-screening stage. A total of 36 videos were examined by all assessors. With the agreement of all of them, 15 videos were excluded for the following reasons: two videos were in a different language than English, nine videos were duplicates, and four videos were about the ESP block performed with a landmark technique. A total of 21 videos entered the final analysis.

The technical quality resulted in 16 unsatisfactory videos (76.1%), one poor video (4.8%), three satisfactory videos (14.3%), one good video (4.8%) and zero outstanding videos (0%). The video quality resulted in eight unsatisfactory videos (38%), five poor videos (23.8%), five satisfactory videos (23.8%), two good videos (9.6%) and one outstanding video (4.8%). Data are summarized in Table 3.

**Table 3:** Overview of the data Video links are reported in the appendices. s: seconds, n: numbers

Video number	Academic	Duration(s)	Views(n)	Quality -1	Quality-2
1	Yes	1102	58347	46	61
2	No	135	5409	8	37
3	Yes	697	10429	13	32
4	No	347	324	1	17
5	No	191	27452	35	53
6	Yes	191	631	7	33
7	No	149	8920	0	4
8	Yes	56	10736	6	17
9	No	85	711	13	22
10	No	140	420	0	2
11	No	235	155	1	6
12	No	193	271	8	35
13	No	219	287	35	48
14	No	153	1695	4	23
15	No	93	325	4	8
16	No	30	59	2	13
17	Yes	462	52	37	37
18	No	80	20	3	5
19	No	15	444	0	0
20	No	72	84	0	0
21	No	84	146	17	21

The academic videos had higher technical quality scores 13(30) vs 3.5(8.5) (*p*-value = 0.047), but no significant difference was recorded in video quality scores 33(5) vs 15(21.25) (*p *= 0.075), video length 462(506) vs 137.5(108.5) (*p*-value = 0.082) and video views 10429(10105) vs 324.5(804.25) (*p*-value = 0.120).

**Figure 1:**
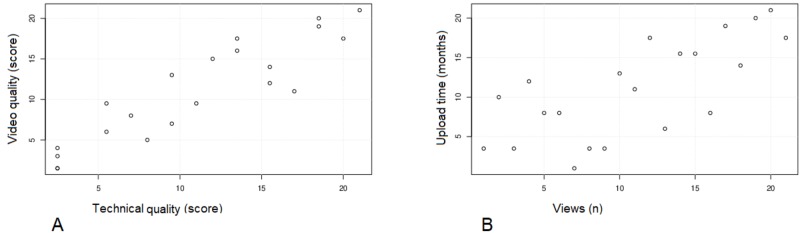
Scatterplot of ranks Panel A: correlation between video and technical quality; panel B: correlation between upload time and views. n: numbers

A strong correlation was found between the technical and video quality (rho = 0.91, *p* < 0.001; Figure 1A) and between upload time and visualizations (rho = 0.72, *p *< 0.001; Figure 1B); a weaker but significant correlation was found between the video length and both technical quality (rho = 0.48, *p *= 0.02) and video quality (rho = 0.62, p = 0.003); no correlation was found between visualizations and both technical (0.23, *p* = 0.323) and video quality (0.35, *p* = 0.118)

Inter-rater reliability estimated by Fleiss’ kappa was moderate for both questionnaires (0.60 for the first questionnaire and 0.68 for the second questionnaire).

## Discussion

Ultrasound-guided interfascial blocks are an emerging topic in anesthesia as safe, easy to perform, and opioid-sparing techniques. The transversus abdominis plane was the first ultrasound-guided interfascial block proposed and, since then, many others have been described such as PECS block, serratus block, and quadratus lumborum block. The ESP block was the last block described in chronological order [10-11].

Recently, a systematic review highlighted how the majority of knowledge about the ESP block originates from case reports and series and not from randomized controlled trials (RCTs) [6]. Despite the lack of RCTs the interest for ESP block is impressive, as this is pointed out by the high number of publication regarding the ESP block in the last two years (one paper every five days) [6]. This huge interest motivated us to investigate the quality level of the educational ESP material available on the Internet. As a matter of fact, the education and training of novice anesthesiologists are of primary importance in order to guarantee quality and safety for patients; this is especially true when a new technique is being developed.

Traditional education consists of handbooks, graduate and postgraduate courses, and tutored practice. However, Internet and video-sharing platforms in the last decades have positioned themselves as important ways to obtain access to updated medical knowledge. In fact, video resources can be more helpful than traditional methods as they can provide at the same time an audio description, a virtual image and the actual image of the technique execution; for this reason, the Internet has become the resource of choice for physicians to follow innovation and to stay up to date in their field of expertise [12-13]. A recent survey conducted on surgeons regarding laparoscopic surgery showed that more than 86% of the trainees routinely watch online surgical videos on YT to learn or perfect the surgical technique [14]. Although several studies have investigated the educational effects of video sharing platforms such as YT its exact role in training has yet to be determined [7-8,13-16].

As stated above, video-sharing platforms as YT have no direct control on the uploaded material, resulting in both high and low-quality videos. Former studies found that the quality of both educational and medical procedures found on YT videos was low [7-8,13-16]. Our study confirms that the overall video quality is really low: 80.9% of the videos had a poor to totally unsatisfactory technical quality and 61.8% of the videos had a poor video quality or worse. Previous studies on anesthesia topics found similar low quality in videos regarding spinal/epidural and brachial plexus procedures [7-8]. Worse than low quality per se some studies showed that these videos could contain incorrect information potentially leading to negative outcomes. [17-20]. In our study we found no formally incorrect information; however, some videos had such a low quality that they could be easily misinterpreted by novice practitioner especially due to low-quality ultrasound images, poor or missing audio comments and unsatisfactory description of the technique.

Although videos uploaded by anesthesia societies and by field experts had higher video and technical quality, video quality was only close to be statistically significant (*p *= 0.075) probably because of the low sample size. Higher academic video quality in those uploaded by anesthesia societies was instead in line with what was already reported by other similar works [21-22]. In fact, if on one side academic videos are made by topic experts, on the other side they undergo an internal review of the contents before being released to the public, thus increasing the quality of the released video.

A final interesting consideration is made regarding the video views. We can imagine video views as the number of physicians looking to learn or to perfect the technique. We found a strong correlation only between video upload time and the number of views for each video, but there was no correlation between quality and views, suggesting that the information contained in low-quality video reaches the same number of users as higher quality academic videos; this finding is concordant with former studies [7,23]. Ideally, the best quality videos should have a higher number of viewers and the lower-quality videos the lowest. However, from our data, we can presume that a physician looking for an educational video on YT has the same chance to find as a first result a high or a low-quality video and to learn the technique from it. This finding raises an issue about YT positioning itself as an educational tool.

Limitations

Our study has some limitations that need to be discussed. We recognize that three assessors could be a low number; however, inter-rater reliability was adequate for both questionnaire. We analyzed only YT as a video-sharing platform, but we acknowledge that several other video-sharing platforms exist. Inter-rater reliability was adequate, however giving more descriptive definition rather than “zero: totally satisfying, one: unsatisfactory, two: poor, three: satisfactory, four: good, five: outstanding” could have led to a higher agreement between assessors.

## Conclusions

Although academic videos have a better quality than non-academic ones, the educational material for the ESP block existing on YT has a limited technical and video quality. For this reason, we recommend physicians to be cautious using video-sharing platforms as a unique source of medical knowledge while learning ESP block.

## Appendices

Links of the videos included in the research

Video links as URL and the last accessed date are provided (Table 4).

**Table 4:** Video links

#	Video Link
1	https://www.youtube.com/watch?v=EVowRjEFUfk (Last accessed 28/02/2019)
2	https://www.youtube.com/watch?v=PRtXJ-7clt0 (Last accessed 28/02/2019)
3	https://www.youtube.com/watch?v=LOCkhD-80EU (Last accessed 28/02/2019)
4	https://www.youtube.com/watch?v=eVaqgAKGVAo (Last accessed 28/02/2019)
5	https://www.youtube.com/watch?v=1YKfTpMeveg (Last accessed 28/02/2019)
6	https://www.youtube.com/watch?v=OHp5V1wVyjA (Last accessed 28/02/2019)
7	https://www.youtube.com/watch?v=w_fd60miMhM (Last accessed 28/02/2019)
8	https://www.youtube.com/watch?v=RYNxeBSDuaA (Last accessed 28/02/2019)
9	https://www.youtube.com/watch?v=VdnH9-Nfi2E (Last accessed 28/02/2019)
10	https://www.youtube.com/watch?v=P0gjck1ASiY (Last accessed 28/02/2019)
11	https://www.youtube.com/watch?v=MD4dJf4wpqk (Last accessed 28/02/2019)
12	https://www.youtube.com/watch?v=5eOtfZa92Os (Last accessed 28/02/2019)
13	https://www.youtube.com/watch?v=bIH3Accg0wQ (Last accessed 28/02/2019)
14	https://www.youtube.com/watch?v=MtgK4JIPhB8 (Last accessed 28/02/2019)
15	https://www.youtube.com/watch?v=arCKiPRzNLk (Last accessed 28/02/2019)
16	https://www.youtube.com/watch?v=3od2Q4e8DfE (Last accessed 28/02/2019)
17	https://www.youtube.com/watch?v=78CyDt9o3Fw (Last accessed 28/02/2019)
18	https://www.youtube.com/watch?v=AIO1xRE1vuA (Last accessed 28/02/2019)
19	htps://www.youtube.com/watch?v=L6jr7MFKWqA (Last accessed 28/02/2019)
20	https://www.youtube.com/watch?v=aayXVrTu5Bs (Last accessed 28/02/2019)
21	https://www.youtube.com/watch?v=KS_cMzU54zs(Last accessed 28/02/2019)
